# Alcohol and HCV Chronic Infection Are Risk Cofactors of Type 2 Diabetes Mellitus for Hepatocellular Carcinoma in Italy

**DOI:** 10.3390/ijerph7041366

**Published:** 2010-03-29

**Authors:** Massimiliano Balbi, Valter Donadon, Michela Ghersetti, Silvia Grazioli, Giovanni Della Valentina, Rita Gardenal, Maria Dal Mas, Pietro Casarin, Giorgio Zanette, Cesare Miranda, Paolo Cimarosti

**Affiliations:** 1 Department of Medicine, Internal Medicine 3^rd^, Pordenone Hospital, Via Montereale 24, Pordenone 33170, Italy; E-Mails: drbalbim@yahoo.it (M.B.); michela.ghersetti@aopn.fvg.it (M.G.); silvia.grazioli@aopn.fvg.it (S.G.); giovanni.dellavalentina@aopn.fvg.it (G.D.V.); rita.gardenal@aopn.fvg.it (R.G.); maria.dalmas@aopn.fvg.it (M.D.M.); pietro.casarin@aopn.fvg.it (P.C.); 2 Diabetes Clinic, Pordenone Hospital, Via Montereale 24, Pordenone 33170, Italy; E-Mails: giorgio.zanette@aopn.fvg.it (G.Z.); cesare.miranda@aopn.fvg.it (C.M.); 3 Community Addictions Service, Pordenone Hospital, Via Montereale 24, Pordenone 33170, Italy; E-Mail: paolo.cimarosti@aopn.fvg.it

**Keywords:** Hepatocellular carcinoma, Type 2 diabetes mellitus, HCV infections, alcohol abuse

## Abstract

Type 2 diabetes mellitus (DM2) has been associated with hepatocellular carcinoma (HCC) development. To study this relationship, we enrolled 465 HCC patients compared with 618 Cirrhotic cases and 490 Controls. The prevalence of DM2 is significantly higher in HCC patients with an Odds Ratio of 3.12 *versus* Controls. In HCC cases with alcohol abuse, the frequency of DM2 is the highest. In our HCC patients, when HCV infection is associated with alcohol abuse, the liver cancer develops earlier. In addition, multivariate analysis shows that alcohol consumption is an independent risk factor for HCC more relevant than HCV infection.

## 1. Introduction

Hepatocellular carcinoma (HCC) is the fifth most common malignancy worldwide and the third leading cause of cancer-related deaths [1]. In recent years, a significant increase in hepatocellular carcinoma incidence and mortality rates has been observed in developed countries, but the causes of this growth are only partially understood. Although the main risk factors for HCC are hepatitis C virus, hepatitis B virus (HBV) and chronic alcohol abuse, at least 25% of HCC cases do not have any known etiology, suggesting that further risk factors could be responsible for the increasing incidence of HCC. Diabetes mellitus has recently been proposed as a risk factor for HCC [2,3]. During the past two decades, the prevalence of diabetes mellitus, and in particular of type 2 diabetes mellitus, has dramatically increased in many countries, including Italy [4]. Sedentary lifestyles, excessive food consumption and obesity appear to be the main causes of the current diabetes mellitus epidemic in the western world [5].

Previous studies on the association between diabetes mellitus and liver diseases showed that type 2 diabetes mellitus appears to be a cause of non-alcoholic fatty liver disease (NAFLD) and that cirrhosis and hepatitis C virus infection increase the susceptibility to diabetes mellitus [6,7].

Moreover, conflicting results were reported on the association between diabetes mellitus and solid tumors, in particular HCC [3,8–12]. While earlier investigations did not report any association between diabetes mellitus and HCC, recent data clearly indicate that diabetes mellitus is a risk factor for HCC [13–15].

However, the precise relation between diabetes mellitus and chronic liver diseases still needs to be further investigated. Two recent studies have suggested a synergistic interaction in the HCC occurrence among heavy alcohol consumption, chronic viral hepatitis and diabetes mellitus in American blacks and whites [16,17]. The aims of our study are to provide a precise assessment on the effect of alcohol consumption and HCV infections, alone and as co-factors of diabetes mellitus, on the risk of HCC in Italian patients.

## 2. Methods

### 2.1. Ethics

This work has been carried out in accordance with the Declaration of Helsinki (2000) of the World Medical Association.

We performed a retrospective, case-control study on three groups of Caucasian individuals, attending the Liver Unit and Diabetic Clinic of 3rd Internal Medicine in the Pordenone General Hospital (Pordenone, Italy) from January 1994 to June 2006. The 3rd Internal Medicine of Pordenone Hospital is a tertiary referral centre for liver disease and diabetes mellitus. This study is a single centre investigation and all patients of the three groups studied were all afferent, directly diagnosed and followed-up in the 3rd Internal Medicine of Pordenone Hospital.

A series of patients with HCC was compared with two different groups: one consisted of patients with liver cirrhosis (LC) and the other one, the Controls, included individuals who were treated in our Hospital for a wide spectrum of acute conditions. The demographic features of these groups are summarized in Table 1.

### 2.2. Sample

#### 2.2.1. HCC group

The HCC group comprised 465 consecutive patients with HCC, of which 398 cases (85.6%) were diagnosed by means of cytological or histological examination of hepatic focal lesions. The others (14.4%) were diagnosed according to the following acknowledged criteria [18]: ultrasound examination (also by using micro-bubbles of sulfur hexafluoride as contrast dye in the last three years), alpha fetoprotein (AFP) > 400 ng/mL, computerized tomography scan and/or magnetic resonance imaging of the upper abdomen. Clinical data, biochemical parameters and the antidiabetic treatment were considered at the time of HCC diagnosis.

#### 2.2.2. LC group

We enrolled 618 patients with liver cirrhosis (LC), matched with the HCC cases by age (±5 years), gender, body mass index (BMI), transaminases, history of diabetes, prevalence of hepatitis B virus and hepatitis C virus infections, alcohol consumption and time of admission. These patients were admitted to our hospital for diagnosis, staging or therapy of liver cirrhosis. Clinical data, biochemical parameters and antidiabetic treatment were considered at the time of recruitment.

According to Child’s classification of cirrhosis, patients were classified as follows: class A: 55.5%; B: 24.3% and C: 20.2%. In the cirrhotic patients, the presence of HCC was ruled out through ultrasound examinations, CT or MRI of the upper abdomen and AFP checks.

#### 2.2.3. Control group

From 28,740 in-patients of our region, 490 subjects matched with HCC and LC patients by age (±5 years), gender, BMI, history of diabetes and time of admission were recruited. Those who were admitted for malignancies, alcohol-related disease, viral liver disease and diabetes mellitus were excluded from our study, although comorbidity of these conditions was not considered as an exclusion criterion. Therefore, the primary diagnoses of admission were: heart failure (34.9%), hypertension (21.4%), chronic obstructive broncho-pneumopathies or pneumonia (16.5%), atrial fibrillation (7.8%), deep venous thrombosis (6.5%), fever of unknown origin (5.3%), benign tumors (4.1%), gastritis (3.5%).

The Control group subjects are not matched with LC and HCC patients for HCV and HBV infection, alcohol consumption, diabetes mellitus history and antidiabetic treatment. In fact, our purpose was to select the Controls in order to be sure that they represent our region’s general population as regards to HCV, HBV infection, alcohol consumption and diabetes mellitus prevalence in subjects of the 60−75 years age group, which is the age interval in which the mean age of our HCC patients is located.

Therefore, the prevalence for the following parameters, as total in the free living population of our region and in their age interval of 60−75 years, compared to those of the selected Controls, are the following:

- HCV infection: 3.2% total and 5.0% in the age group of 60−75 years of the general population as regards to 5.3% of Controls in the present study;
- HBV infection: 1.2 % in all the general population as to 1.68% in the age group of 60−75 years and 1.15 % in Controls;
- Alcohol abuse: 4.5% in general population as to 9.7% in the 60−75 years age group and 9.5% in Controls;
- Diabetes mellitus : 4.8% in the general population as regards to 12.4% in the age group of 60−75 years and 12.7% in Controls of this study [19–22].

### 2.3. Data Collection

The demographic, clinical and biochemical data of each patient were collected in a computerized database. Biochemical parameters were determined at the Pordenone Hospital central laboratory using standardized and validated methods.

Hepatitis B surface antigen (HBsAg), anti-HBV surface antigen (anti-HBs), anti-HBV core antigen (anti-HBc), and hepatitis B e antigen (HBeAg) were determined using commercial assays (Abbott Diagnostic Division, Wiesbaden; Germany). Sera were also screened for antibodies against HCV (anti-HCV) using a third-generation micro particle enzyme immunoassay (AxSYM HCV version 3.0, Abbott Diagnostic Division). Positive samples were tested for anti-HCV using a third-generation line immunoassay (Immunogenetics, Gent, Belgium) and for serum HCV-RNA using the Roche Amplicor version 2.0 (Roche Molecular System, Pleasenton, CA).

The diagnosis and clinical classification of diabetes mellitus were based on the guidelines of the American Diabetes Association [23,24]. In particular, the distinction between type 1 and 2 diabetes mellitus, was made according to the following clinical characteristics: age and modality of glucose intolerance onset, previous use of antidiabetic medications, occurrence of ketoacidosis, obesity and body fat distribution, concomitant autoimmunity, positive family history and HLA association, presence of micro- and macrovascular complications when diabetes was diagnosed.

A trained nurse assessed the alcohol intake by interviewing the patients using a standard questionnaire. Total alcohol intake was evaluated retrospectively on the basis of a history of lifetime consumption, dividing the patient’s life into 10-years periods, and recorded as the average amount of ethanol (mL) ingested daily. Each subject was classified according to his/her maximum level of alcohol consumption for one or more decades in his/her lifetime in order to avoid underestimation of alcohol consumption in subjects with HCC and a history of alcoholic liver disease. Alcohol use was evaluated considering an average alcohol content in volume of 5% for beer (a can = 330 mL), 12% for wine (a glass = 125 mL), 18% for aperitifs, 30% for digestive alcoholic drinks and 40% for liqueurs (a measure = 40 mL). A glass of wine, a can of beer or a measure of superalcoholics contain about 16 g of ethanol. Alcohol abuse was defined as a daily consumption over 30 g for males and over 20 g for females. Average alcohol content was estimated in 5% for beer, 12% wine and 40% spirits [25].

### 2.4. Statistical Analysis

Normality tests were performed on all data. Parametric data are expressed as mean values ± standard deviation (SD). Data with multiple time points variables were analysed by the general model ANOVA. *Post hoc* multiple comparisons were performed using an LSD test when ANOVA testing was significant (*P* ≤ 0.05). To establish univariate associations among variables, the odds ratio (OR) with a confidence interval of 95% was calculated, using the simple analysis of the logistic regression. Multivariate logistic regression was used to assess the association of HCC with DM2 after adjusting for potentially confounding factors. Evaluating the association of DM2 with the risk of HCC using LC patients as the Control group, we considered sex, age, body mass index, HBV and HCV infection, alcohol abuse, ALT level. Using the Control group, potential confounders included only sex, age, BMI, and alcohol abuse. We examined the potential interaction effects between the independent risk factors on HCC risk. When assessing if the combined effect of two factors on HCC was greater than the sum of the individual effects, we used the Synergy Index (SI) method described by Rothman [26]. A value of SI equal to unity was interpreted as indicative of additivity, whereas a value greater than unity was indicative of superadditivity and synergism.

All statistical analyses were performed using SAS v9.1 (SAS Institute Inc., Cary, NC, USA).

## 3. Results

Each subject with diabetes mellitus in the HCC and LC groups showed the clinical and metabolic characteristics of type 2 diabetes mellitus. Of note, none of our HCC or liver cirrhosis patients had type 1 diabetes mellitus.

As shown in Table 2, the prevalence of DM2 was 31.2% in the HCC group, 23.3% in the LC group and 12.7% in the Controls. The prevalence of DM2 in the three groups were statistically different (*P* < 0.0001), with an OR of 3.12 (CI 2.2−4.4; *P* < 0.001) in HCC group *versus* Controls and an OR of 2.09 (CI 1.5−2.9; *P* < 0.001) in HCC *versus* LC cases.

Etiology of the chronic liver disease, mean age at HCC diagnosis and DM2 prevalence in the HCC patients are summarized in Table 3. Notably, alcohol related HCC had a significantly higher prevalence of DM2 compared to the HCV-positive group of HCC (36.9% *versus* 26.5%; *P* = 0.048).

### 3.1. Age at Diagnosis of HCC According to Etiology of Liver Disease

In the subgroup of patients with HCV infection associated to alcohol abuse, the age of HCC diagnosis is similar to the patients with only alcohol abuse as a risk factor for HCC (66.7 ± 8.5 years), but significantly lower than in the subgroup of patients with HCV infection (67.7 ± 9.3 *versus* 71.5 ± 7.3 years; *P* < 0.001) (Table 3).

### 3.2. Multivariate Analysis

Multivariate logistic regression analysis was used to assess the independent role of different variables in HCC patients considering Controls as the comparison group (Table 4). HCV infection, alcohol abuse and DM2 are independent variables associated with risk of HCC and alcohol abuse shows the highest risk of HCC occurrence (OR = 121.2; CI 61.9–233.7; *P* < 0.001).When considering cirrhotic patients as comparison groups the OR for DM2 was 1.4 (CI 1.0–1.9; *P* = 0.01).

### 3.3. Time Interval from Type 2 Diabetes Mellitus Onset to HCC Diagnosis

The data collected in the records of our Diabetes Clinic show that in 122 patients (84.9%), DM2 was diagnosed at least six months before the onset of HCC, while in only 23 patients (15.1%) DM2 was recognized after the diagnosis of HCC.

The time interval between DM2 diagnosis and HCC onset was exactly calculated: diabetes was found to be present prior to the HCC diagnosis for a mean time of 141.5 ± 9.4 months.

In the subgroup with pre-existing DM2, the time interval until the diagnosis of HCC was longer in insulin treated patients than in those treated with antidiabetic oral agents (171.5 ± 87.6 *versus* 118.7 ± 95.2 months; *P* < 0.05) (Table 5).

### 3.4. Interactive Effect of the Association among Type 2 Diabetes Mellitus and HCV Infection

As regards to the effect of HCV as cofactor of DM2, we found an *OR* for HCC of 2.7 (CI 1.9−4.0; *P <* 0.0001) in HCC patients *versus* Controls with HCV infection alone and of 36.9 (CI 21.6−60.5; *P <* 0.0001) if HCV infection is associated with DM2, with a Rothman’s Synergy Index of 9.2. (CI 4.7−17.2) (Table 6).

### 3.5. Interactive Effect of the Association among Type 2 Diabetes Mellitus and Alcohol Abuse

Evaluating the association among DM2 and alcohol abuse in HCC *versus* Controls group, the alcohol abuse alone shows an *OR* for HCC of 3.7 (CI 2.5−5.4; *P <* 0.0001) and of 49.0 (CI 21.5−111.8; *P <* 0.0001) when alcohol is associated to DM2, with a Rothman’s Synergy Index of 9.8 (CI 4.9−18.4) (Table 7).

## 4. Discussion

Our study shows that HCV infection, alcohol abuse and DM2 are independent risk factors for HCC. Alcohol abuse seems to have a stronger effect on HCC risk, more than HCV infection and DM2 by itself. In fact, the *OR* for HCC is significantly higher when DM2 is associated with alcohol abuse (49.0) than with chronic HCV infection (36.9). In addition, alcohol consumption significantly lowers the mean age of HCC occurrence in HCV-positive patients of our population and the prevalence of DM2 that is higher in patients with alcohol abuse than in HCV-positive patients.

In this study, every HCC and cirrhotic patient with abnormal glucose tolerance showed clinical and pathophysiological characteristics of DM2. The results of our study are consistent with the theory of the biological mechanisms underlying the epidemiological association between diabetes mellitus and cancer. In fact, this hypothesis postulates that the diabetes-cancer association is likely to be related to insulin resistance and consequent hyperinsulinemia, which are typical features in the majority of type 2 diabetes mellitus patients. Ten years ago, McKeown-Eyssen [27] and Giovannucci [28] observed that risk factors for cancer and insulin resistance in developed countries are almost the same. To explain this analogy, they suggested that protracted exposure to hyperinsulinemia increases the levels of IGF-1, which plays a pivotal role in carcinogenesis (insulin-cancer hypothesis) [29]. In addition, the predictive value of hyperinsulinemia on total cancer mortality [9] and fatal liver tumor incidence [30] has been demonstrated in non-diabetic subjects by two recent prospective analysis.

The mechanisms through which alcohol consumption and diabetes mellitus promote the development of HCC are unknown. Although moderate alcohol intake is associated with a reduced risk of DM2, high alcohol consumption seems to have an increased risk of DM2 compared to moderate consumers [31]. There is a supposed role of oxidative stress, like during the heavy alcohol consumption, in the onset and progression of diabetes mellitus and its complications, related to chronic hyperglycemia. In diabetes mellitus patients, an increased blood glucose level may stimulate glycosylation of proteins, including hemoglobin, leading to an increase in the release of iron from hemoglobin and further production of free radicals causing oxidative stress, in fact the finding of high concentration of serum ferritin in patients with diabetes mellitus may reflects an increased body iron stores [32–36]. The fact that iron is a powerful pro-oxidant and that oxidative stress is increased in impaired glucose tolerance states, suggests a possible role for oxidative stress in pathogenesis of diabetes mellitus and its complications on the liver, such as cirrhosis. It is also possible that alcohol-induced oxidative stress may increase the susceptibility of patients with diabetes mellitus to DNA damage and HCC development. Moreover, heavy alcohol consumption could directly induce hepatic cellular injury and toxicity leading to the development of liver fibrosis and cirrhosis [37]. In addition, oxidation and metabolism of ethanol in the liver by microsomal enzymes may contribute to liver carcinogenesis [38].Generation of acetaldehyde, and oxygen free radical during ethanol metabolism is also associated with development of alcohol related liver disease through oxidative stress [39].

The association between chronic ethanol abuse and the development of cirrhosis, as well between cirrhosis and the development of HCC is well documented. Ethanol and its metabolism affect cell signaling pathways that regulate normal and abnormal hepatocyte function, proliferation and apoptosis, deplete the body’s natural antioxidant supply of glutathione and increase oxygen radical production, leading to release of proinflammatory cytokines [40]. Oxidative stress is a central factor involved in alcohol-induced liver injury and a critical event in carcinogenesis. [41]. Moreover intake of alcohol results in decreased hepatic levels of vitamin A, that plays an important role in controlling cell growth, differentiation and apoptosis, as well on carcinogenesis [42].

In type 2 diabetes mellitus patients the mortality ratio for liver related events is higher than that for cardiovascular events [43]. However, patients with DM2 often suffer from liver disease, and diabetes is a recognized cause of NAFLD and cryptogenic cirrhosis. In fact, it is well-known that the natural history of NAFLD might progress, over a period of many years, from steatosis to steatohepatitis, cirrhosis and, sometimes, to HCC [6]. On the other hand, 20% of patients with cirrhosis have overt diabetes (hepatogenous diabetes) and 60% have impaired glucose tolerance [44]. Thus, the association between diabetes and cirrhosis is complex and reciprocal.

To evaluate the interactions between DM2, HCV infection, alcohol abuse and HCC, we performed a single centre, retrospective case-control study on HCC patients comparing them, not only to a group of Control subjects without liver diseases and diabetes mellitus, but also to a series of cirrhotic patients.

Our study shows that the prevalence of DM2 in the LC group is intermediate between those of the HCC and Controls, indicating that the underlying liver cirrhosis is not the only cause of diabetes in HCC patients. The evidence that DM2, HCV infections and alcohol abuse are a risk factor for HCC in our patients is demonstrated by multivariate analyses, which show an OR for HCC of 2.2 for DM2, 106.5 for HCV infections and 121.2 for alcohol abuse. The prevalence of DM2 is significantly higher in HCC patients with alcohol abuse and have a synergic effect on HCC occurrence more evident than the association between DM2 and HCV infection.

The precise temporal relation between the onset of type 2 diabetes mellitus and diagnosis of HCC is only partially understood. A previous prospective study [11], conducted in a large cohort of males with and without diabetes mellitus, investigated for the first time, the temporal relationship between diabetes and HCC, showing a two-fold increase of HCC incidence among patients with diabetes. Our study showed that DM2 is present for a mean of 141 months before the diagnosis of HCC.

A new finding of the current study is the demonstration of synergistic (excess over additivity) effects on HCC between HCV infections, alcohol abuse and diabetes, independent of each other’s effects. Indeed the combined effect of viral hepatitis C and diabetes and alcohol abuse and diabetes were both more than the sum of the individual effects as showed by the Rothman’s Synergy Index that is more relevant for alcohol abuse than for HCV infection as cofactors of diabetes mellitus on HCC development. This suggest that heavy alcohol consumption, in addition to its own direct effects may exacerbate the effect of diabetes mellitus and chronic hepatitis C virus infection on chronic liver diseases and HCC.

## 5. Conclusions

The results of our study, therefore, have important implications in the clinical management of diabetes mellitus, particularly in patients with DM2 and chronic liver diseases, because we found that they are at high-risk for HCC. This observation is of primary relevance to the implementation of prevention policies and to encourage the most adequate and cost-effective programs of surveillance in cirrhotic patients.

Thus, our data suggests that patients with DM2 and chronic liver disease should first control their diabetes through diet and changes in lifestyle, to decrease their weight and increase physical activity.

In our study HCV infection, alcohol abuse and DM2 are independent predictors of HCC development. Furthermore our data show that in our population, alcohol consumption, DM2 and HCV-positivity exert synergistic effects on risk of HCC. These factors are likely contributors to the rising incidence of HCC in our and other developed countries. Moreover, public health considerations should prompt the study of HCC prevention strategies among high-risk individuals. In the meantime, it would seem prudent for patients having chronic hepatitis infections or diabetes mellitus consider abstaining from alcohol consumption to reduce the effect of alcohol when combined with these other risk factors.

## Figures and Tables

**Table 1.** Characteristics of the HCC, LC and Control groups.

	**HCC (n = 465)**	**LC (n = 618)**	**Controls (n = 490)**	***P***
**Sex** Male n (%)	364 (78.3)	450 (72.8)	385 (78.6)	NS^1^
**Age** years mean ± SD	68.5 ± 8.9	64.9 ± 12.2	69.4 ± 13.8	NS^1^
**BMI** kg/m^2^ mean ± SD	25.2 ± 3.1	25.1 ± 3.2	25.1 ± 2.9	NS^1^
**Alcohol abuse** n (%)	141 (30.4)	188 (30.5)	46 (9.5)	NS^2^
**DM2** n (%)	145 (31.2)	144 (23.3)	62 (12.7)	<0.001^1^
**HBV** + n (%)	20 (4.3)	27 (4.4)	5 (1.1)	NS^2^
**HCV** + n (%)	177 (38.1)	236 (38.3)	26 (5.3)	NS^2^
Duration of type 2 diabetes months, mean ± SD	141.5 ± 9.4	140 ± 10.5	N/A	NS^2^
Duration of cirrhosis Months, mean ± SD	150.2 ± 11.6	144.3 ± 9.6	N/A	0.01
Child-Pugh score (A, B, C %)	55.5, 24.3, 20.2	69.3, 22.2, 8.5	N/A	

Legend: HCC = hepatocellular carcinoma, LC = liver cirrhosis, SD = standard deviation, BMI = body mass index, DM2 = type 2 diabetes mellitus, N/A = not available, NS = not significant

1 *P*-value of chi-square test

2 *P*-value of chi-square test for HCC *versus* LC group.

**Table 2.** Frequency of type 2 diabetes mellitus in HCC, LC and control groups.

**Subjects (*n*)**	**DM2-negative *n* (%)**	**DM2-positive *n* (%)**	***OR* (95% CI)**	***P***

**Total**				
**-HCC**(465)	320 (68.8)	145 (31.2)	3.12 (2.2–4.4)^a^	<0.001
**Controls** (490)	428 (87.3)	62 (12.7)	2.09 (1.5–2.9)^c^	<0.001
**- LC** (618)	474 (76.7)	144 (23.3)		

**Males**				
**- HCC** (364)	246 (67.6)	118 (32.4)	3.14 (2.1–4.6)^b^	<0.00001
**- Controls** (385)	334 (86.7)	51 (13.3)	1.99 (1.3–2.9)^d^	=0.0002
**- LC** (450)	341 (75.8)	109 (24.2)		

**Females**				
**- HCC** (101)	74 (73.3)	27 (26.7)	3.11 (1.3–7.4)^e^	=0.002
**- Controls** (105)	94 (89.5)	11 (10.5)	2.59 (1.2–5.9)^f^	=0.008
**- LC** (168)	133(79.2)	35 (20.8)		

*OR*: Odds ratio; CI: Confidence interval;

a HCC *versus* Controls;

C HCC *versus* LC;

b HCC *versus* Controls;

d HCC *versus* LC;

e HCC *versus* Controls;

f HCC *versus* LC.

**Table 3.** Etiology, mean age and prevalence of type 2 diabetes mellitus in 465 HCC patients. (mean ± SD).

**Etiology**	**HCC *n* (%)**	**Age (yr)**	**Prevalence of DM2 (%)**
**HBV**	20 (4.3)	63.3 ± 10.3^a^,^b^	3 (15.0)
**HCV**	177 (38.1)	71.5 ± 7.3 a,b,c,d,e	47 (26.5)^f^
**Alcohol**	141 (30.4)	66.7 ± 8.5 e	52 (36.9)^f^
**HBV+HCV**	8 (1.7)	60.8 ± 12.8 a	2 (25.0)
**HBV+alcohol**	9 (1.9)	62.9 ± 9.3 a,c	2 (22.2)
**HCV+alcohol**	81 (17.4)	67.7 ± 9.3 a,d	27 (33.3)
**HBV+HCV+alcohol**	2 (0.4)	68.4 ± 10.3	0
**Cryptogenic**	27 (5.8)	68.6 ± 9.3	19 (70.3)

a *P* < 0.001;

b *P* < 0.001;

c *P* < 0.001;

d,e *P* < 0.001;

f *P* = 0.048.

**Table 4.** Multivariate analysis for HCC risk.

HCC	*OR (95% CI)*	*P*
DM2-positive	2.2 (1.2–4)	=0.011
HCV-positive	106.5 (58.2–194.9)	<0.001
ALCOHOL-positive	121.2 (61.9–233.7)	<0.001

*OR*: Odds ratio; CI: Confidence interval.

**Table 5.** Duration of DM2 before HCC diagnosis and antidiabetic therapy.

HCC	Months (mean ± SD)	*P*
Insulin therapy	171.5 ± 87.6	<0.05
AOA therapy	118.7 ± 95.2	
Total	141.5 ± 9.4	

SD: standard deviation.

**Table 6.** Interactive effect of the association of type 2 Diabetes Mellitus with HCV infection in HCC *versus* Controls group.

HCV	DM2	*OR (95% CI)*	*P*	*SI*
No	No	1.0		
Yes	No	2.7 (1.9–4.0)	<0.0001	
Yes	Yes	36.9 (21.6–60.5)	<0.0001	9.2 (4.7–17.2)

*OR*: Odds ratio; CI: Confidence interval; *SI*: Rothman’s Sinergy Index.

**Table 7.** Interactive effect of the association of type 2 Diabetes Mellitus with Alcohol abuse in HCC *versus* Controls group.

Alcohol	DM2	*OR (95% CI)*	*P*	*SI*
No	No	1.0		
Yes	No	3.7 (2.5–5.4)	<0.0001	
Yes	Yes	49.0 (21.5–111.8)	<0.0001	9.8 (4.9–18.4)

*OR*: Odds ratio; CI: Confidence interval; *SI*: Rothman’s Sinergy Index.
